# Modulation of Glial Function in Health, Aging, and Neurodegenerative Disease

**DOI:** 10.3389/fncel.2021.718324

**Published:** 2021-08-31

**Authors:** Kendra L. Hanslik, Kaitlyn M. Marino, Tyler K. Ulland

**Affiliations:** ^1^Neuroscience Training Program, University of Wisconsin, Madison, WI, United States; ^2^Department of Pathology and Laboratory Medicine, University of Wisconsin, Madison, WI, United States

**Keywords:** astrocytes, microglia, aging, neurodegeneration, glia, Alzheimer’s disease, microbiome, Parkinson’s disease

## Abstract

In the central nervous system (CNS), glial cells, such as microglia and astrocytes, are normally associated with support roles including contributions to energy metabolism, synaptic plasticity, and ion homeostasis. In addition to providing support for neurons, microglia and astrocytes function as the resident immune cells in the brain. The glial function is impacted by multiple aspects including aging and local CNS changes caused by neurodegeneration. During aging, microglia and astrocytes display alterations in their homeostatic functions. For example, aged microglia and astrocytes exhibit impairments in the lysosome and mitochondrial function as well as in their regulation of synaptic plasticity. Recent evidence suggests that glia can also alter the pathology associated with many neurodegenerative disorders including Alzheimer’s disease (AD) and Parkinson’s disease (PD). Shifts in the microbiome can impact glial function as well. Disruptions in the microbiome can lead to aberrant microglial and astrocytic reactivity, which can contribute to an exacerbation of disease and neuronal dysfunction. In this review, we will discuss the normal physiological functions of microglia and astrocytes, summarize novel findings highlighting the role of glia in aging and neurodegenerative diseases, and examine the contribution of microglia and astrocytes to disease progression.

## Introduction

The majority of cells that comprise the central nervous system (CNS) lie within two broad classifications: neurons and glia. Glial cells can further be broken down into subtypes including microglia and macroglia, with the latter group consisting of astrocytes, oligodendrocytes, and ependymal cells. Unlike microglia, which originate from the mesoderm and are derived from primitive macrophages in the yolk sac, macroglia have a common neuroectodermal embryonic origin (Curtis et al., 1988; Takahashi et al., 1989; Kaur et al., 2001; Butt, 2009; Ginhoux et al., 2010; Gomez Perdiguero et al., 2015). Among these glial subpopulations, microglia and astrocytes serve as crucial regulators of the innate immune response in the brain (Aloisi, 1999; Raivich et al., 1999; Olson and Miller, 2004; Jack et al., 2005; Choi et al., 2014). Microglia provide a variety of support roles including: synaptic pruning and remodeling in development, parenchymal surveillance to clear metabolic products and deteriorated tissue components in the normal CNS, and degrading pathogenic substances such as amyloid-β (Aβ) plaques or harmful viruses and bacteria in diseased states (Nimmerjahn et al., 2005; Lim et al., 2013; Heneka et al., 2015; Weinhard et al., 2018; Cangalaya et al., 2020; Favuzzi et al., 2021). Additionally, microglia play important roles in regulating myelination, controlling and maintaining vascular integrity, neurogenesis, and astrogliogenesis (Antony et al., 2011; Lampron et al., 2015; Halder and Milner, 2019; Diaz-Aparicio et al., 2020; Dudiki et al., 2020; Hughes and Appel, 2020). Like microglia, astrocytes are immunocompetent, possessing the capability to detect danger signals, responding through the release of chemokines and cytokines, and mounting an immune response (Cornet et al., 2000; Gimsa et al., 2013; Wang et al., 2021). Astrocytes also execute essential functions regulating synaptogenesis, providing metabolic support to neurons, and supporting the integrity of the blood-brain barrier in development and in the normal CNS (Pellerin and Magistretti, 1994; Isobe et al., 1996; Kucukdereli et al., 2011; Sultan et al., 2015; Heithoff et al., 2020). Moreover, astrocytes demonstrate the ability to regulate Aβ as well as α-synuclein (α-syn) pathology and maintain synaptic integrity in neurodegenerative disease (Morales et al., 2017; Katsouri et al., 2020; Saha et al., 2020; Tsunemi et al., 2020).

Previous findings reveal that glial cells are heterogenous, varying across species as well as regionally and spatially within the brain (Lawson et al., 1990; Ko et al., 2013; Herculano-Houzel, 2014). Similarly, glia within the same brain can be differentially reactive or functionally distinct depending on their microenvironment (Lawson et al., 1990; Ko et al., 2013; Boisvert et al., 2018; Lanjakornsiripan et al., 2018). Aging also impacts glia, hindering their normal physiological functions. For instance, microglia in aged mice have a slower, less robust response to insults while aging astrocytes display increased mitochondrial oxidative metabolism that disrupts the cell’s ability to provide neuronal support (Hefendehl et al., 2014; Jiang and Cadenas, 2014). Just as aging impacts glial function, it also affects the composition of one’s microbiome and systemic metabolism, which can, in turn, modulate glial cell activity and contribute to age-associated diseases (Fukagawa et al., 1990; Vogt et al., 2017, 2018; Rash et al., 2018; Spencer et al., 2019; Xu et al., 2019; Lee et al., 2020; Wilmanski et al., 2021).

Furthermore, glial cells have been shown to impact the pathology of neurodegenerative diseases though their roles are complex and can be both detrimental and beneficial. In the context of Alzheimer’s disease (AD), microglial activation has been shown to precede amyloid-β (Aβ) plaque deposition and the formation of tau tangles and may result in enhanced pathology (Yoshiyama et al., 2007; Heneka et al., 2013; Wright et al., 2013; Leyns et al., 2017; Venegas et al., 2017; Ising et al., 2019; Shippy et al., 2020). In contrast, *in vivo* and *in vitro* studies using AD mice and human samples show that microglia are instrumental for Aβ plaque clearance, which could slow AD progression (Wang et al., 2015, 2016; Richter et al., 2020; Zhou et al., 2020). The role of astrocytes in AD pathology is also very complex but not fully understood to date. In an AD mouse model, the inhibition or deletion of astrocyte-specific Stat3, a transcription factor that is required for astrogliosis, ameliorated AD pathology, reducing plaque load and dystrophic or deteriorating neurites (Reichenbach et al., 2019). This study suggests that astrocytic Stat3 signaling could exacerbate AD progression. Conversely, *in vivo* and *in vitro* studies using AD mice or astrocyte-neuron co-cultures illustrate that astrocytes play a neuroprotective role in AD as in their absence, Aβ oligomers were more likely to bind dendrites and result in synaptotoxicity (Pitt et al., 2017; Katsouri et al., 2020). Additionally, there was enhanced Aβ aggregation, synapse deterioration, and memory loss without astrocyte signaling, which would increase AD pathogenicity (Pitt et al., 2017; Katsouri et al., 2020). Astrocytes also appear to play a similarly multifaceted role in the context of other neurodegenerative diseases such as Parkinson’s disease (PD), addressed later in this review.

Here, we discuss current literature addressing the physiological function of microglia and astrocytes. We also review how microglial and astrocyte functions are altered by changes associated with aging and microbiome alterations as well as assess each cell’s contribution to the progression of neurodegenerative diseases. Since mice serve as a primary model for many aspects of neurobiology, this review will primarily focus on data from mouse models and will address how those findings confirm or complement studies in humans.

## Homeostatic Roles of Microglia and Astrocytes in Health

**Figure 1:**
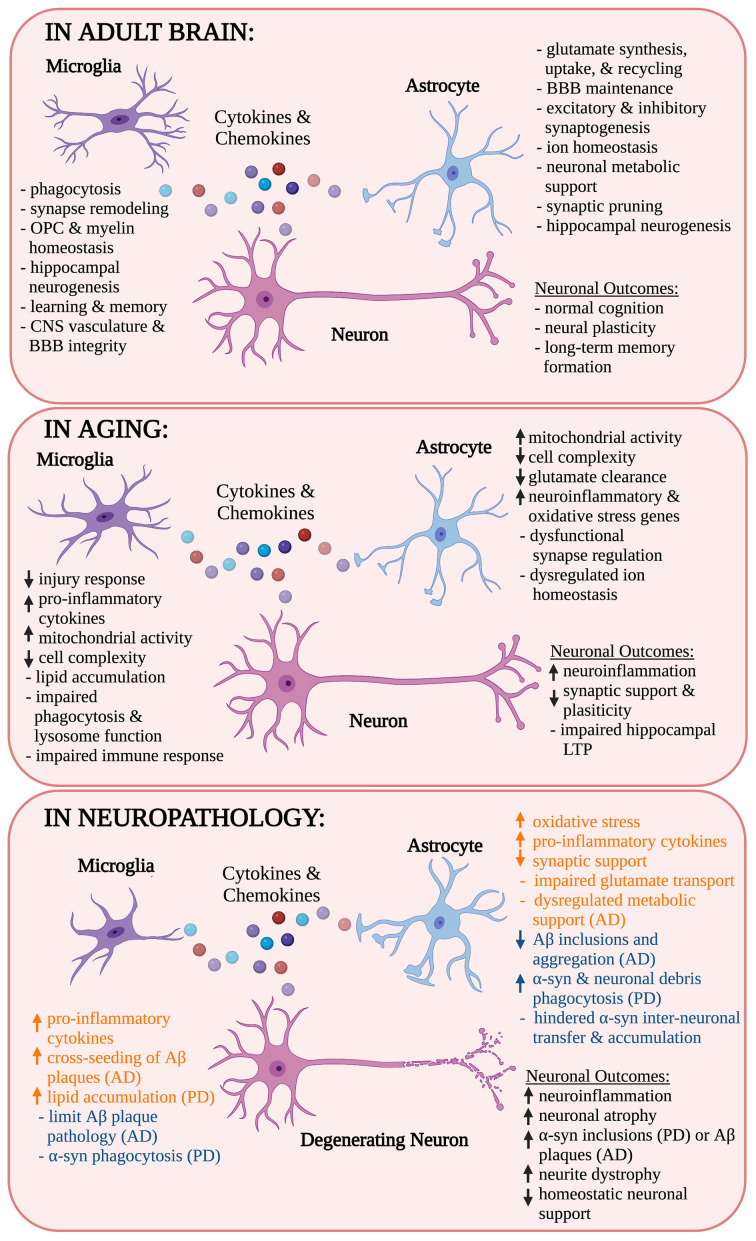
Role of microglia and astrocytes in health, age, and neurodegenerative disease. Microglia and astrocytes release cytokines and chemokines in the neuronal milieu, adopting different responses depending on their surrounding environment. Their responses modulate each other’s activity as well as the activity of neurons and other nearby cell types. In this schematic, the contributions of microglia and astrocytes as well as their influence on neuronal outcomes are identified in health, aging, and neurogenerative disease. Microglial and astrocyte influences in neuropathology are also indicated as pathological (orange) or neuroprotective (blue). OPC, oligodendrocyte progenitor cells; BBB, blood-brain barrier; CNS, central nervous system; LTP, long-term potentiation; Aβ, amyloid-β; AD, Alzheimer’s disease; α-syn, α-synuclein; PD, Parkinson’s disease. *Created with Biorender.com.

Among the glial cells in the CNS, microglia, and astrocytes are primarily responsible for mounting an immune response through the production of cytokines and chemokines (Aloisi, 1999; Raivich et al., 1999; Cornet et al., 2000). In an effort to maintain or return to homeostasis, microglia and astrocytes can repair damage after injury and fight off infections (Figure 1; Myer, 2006; Rasley et al., 2006; Choi et al., 2020; Moseman et al., 2020; Sariol et al., 2020).

Microglia continuously sample their microenvironments within the brain parenchyma by extending and retracting their processes, detecting complement fragments, immunoglobulins, and other inflammatory stimuli e.g., cytokines, chemokines, danger-associated molecular patterns (DAMPs), and pathogen-associated molecular patterns (PAMPs; Raivich et al., 1999; Nimmerjahn et al., 2005). Microglia detect DAMPs and PAMPs using pattern recognition receptors (PRRs) including toll-like receptors (TLRs), nucleotide-binding and oligomerization domain (NOD)-like receptors (NLRs), and absent in melanoma 2 (AIM2)-like receptors among others (Olson and Miller, 2004; Freeman et al., 2017; Ma et al., 2021). Following detection of a DAMP or PAMP, microglia produce and release an array of cytokines and chemokines and become reactive, allowing them to respond to their microenvironment following injury or pathological events (Figure 1; Hammond et al., 2019; Masuda et al., 2019; Wang et al., 2021). In addition to their immune functions, microglia play other key homeostatic roles in the healthy adult brain. First and foremost, microglia serve as the brain’s primary phagocytes, removing dead cells, cell debris, and other harmful, inflammatory stimuli in the healthy brain (Nimmerjahn et al., 2005; Damisah et al., 2020). In adulthood microglial phagocytosis is critical for the removal of new granule cells and the maintenance of long-term hippocampal neurogenesis (Sierra et al., 2010; Diaz-Aparicio et al., 2020). Microglial phagocytosis is also important after brain injury for the removal of cell debris, which prevents secondary neuronal death and reduces additional brain atrophy (Herzog et al., 2019; Wen et al., 2020). Furthermore, microglia modulate neuronal plasticity by monitoring and pruning synapses in the adult brain just as they do in development (Tremblay et al., 2010; Schafer et al., 2012; Ji et al., 2013; Miyamoto et al., 2016; Weinhard et al., 2018). Microglial regulation of synapses can facilitate adaptation to the environment by promoting learning through memory synapse formation or remodeling (Ehninger, 2003; Parkhurst et al., 2013; Nguyen et al., 2020; Wang et al., 2020). Microglia are also active near the brain’s vasculature and are important for maintaining CNS vascular and blood-brain barrier integrity, contributing to the perivascular glia limitans, and regulating fine-scale vasculature remodeling (Lassmann et al., 1991; Halder and Milner, 2019; Haruwaka et al., 2019; Joost et al., 2019; Mondo et al., 2020). More recently, microglia have been cited for playing roles in regulating oligodendrocyte progenitor cell differentiation and myelin homeostasis (Hagemeyer et al., 2017).

Astrocytes also play important immune and homeostatic roles including maintaining ion levels, regulating synaptogenesis, and providing metabolic support to neurons among others (Pellerin and Magistretti, 1994; Kucukdereli et al., 2011). Astrocytes interact with their microenvironment in ways analogous to microglia and express TLRs allowing them to detect tissue injuries, bacteria, viruses, or other pathogenic agents including bacterial products (Bowman et al., 2003; Park et al., 2006). Upon TLR engagement, astrocytes, like microglia, produce and release cytokines and chemokines that serve as mediators of cell migration and communication (Jack et al., 2005; Cordiglieri and Farina, 2010; Choi et al., 2014). Astrocytes are also responsible for glutamate synthesis, uptake, and recycling, a function that has been shown to protect neurons from glutamate-induced excitotoxicity (Shank et al., 1985; Rothstein et al., 1996). Similar to microglia, astrocytes are involved in synaptic pruning and regulating hippocampal neurogenesis, two roles that are both crucial for maintaining circuit connectivity and neural plasticity (Song et al., 2002; Wilhelmsson et al., 2012, 2019; Chung et al., 2013; Magnusson et al., 2020; Lee et al., 2021). Furthermore, astrocytes play a role in maintaining the integrity of the blood-brain barrier (BBB), though this finding has been disputed in some disease contexts (Eugenin et al., 2011; Heithoff et al., 2020). Astrocyte-secreted factors such as hevin, a synaptic glycoprotein, and secreted protein acidic and rich in cysteine (SPARC), an antagonist of hevin’s synaptogenic function, are crucial in excitatory and inhibitory synaptogenesis, which implicates their role in neural plasticity and circuit formation (Hughes et al., 2010; Kucukdereli et al., 2011). Additionally, to ensure the metabolic needs of neurons are met, astrocytes provide them with energetic support through direct astrocyte-neuron metabolic-coupling interactions that can impact long–term memory formation and have neuroprotective effects (Pellerin and Magistretti, 1994; Suzuki et al., 2011; Ioannou et al., 2019).

While microglia and astrocytes each have important functions, these cells do not operate in isolation. Recently, an emerging role for the interplay between astrocytes and microglia in brain homeostasis has been demonstrated, though these examples are not extensive due to technical limitations (Tremblay et al., 2010; Pascual et al., 2012; Damisah et al., 2020). Much of the current knowledge presented about the microglia-astrocyte-neuron interactions have developed through the collective knowledge of how particular cellular crosstalk impacts a cell type, involving considerable speculation, and not through real-time observation (Cerbai et al., 2012; Lian et al., 2016; Liddelow et al., 2017; Shi et al., 2020; Xie et al., 2020). However, using a novel 2Phatal technique, which enables observation of the kinetics of cell death and engulfment through live imaging *in vivo*, distinct roles for microglia and astrocytes were illustrated in neuronal corpse removal in the adult mouse (Damisah et al., 2020). Over various modes of cell death, microglia engulfed cell bodies and proximal dendrites while astrocytes preferentially engulfed distal processes and diffuse neuritic debris during early postnatal development (Damisah et al., 2020). This method also showed that apoptotic cell corpse removal was markedly delayed in 26-month-old aged mice compared to 4-month-old adult mice, which is suggestive of altered glial function in aging (Damisah et al., 2020).

## Modulation of Microglia and Astrocyte Function in Aging

As the brain ages, it is known that cellular and molecular processes are altered causing mitochondrial dysfunction, higher levels of oxidative stress and damage, dysfunctional autophagy mechanisms, and dysregulated stress and inflammatory responses (Mattson and Arumugam, 2018). The normal physiological functions and support roles provided by microglia and astrocytes are also vulnerable to aging. Microglial immune responses following an intraperitoneal injection of lipopolysaccharide (LPS) differ in mice considered middle aged (9–15 months), suggesting that the central nervous system (CNS) may be more vulnerable to age-dependent changes earlier than initially thought (Nikodemova et al., 2016; Keane et al., 2021). Thus, observing how microglial and astrocytic responses change in age is imperative for understanding their contribution to age-associated pathologies.

Aging microglia in both mice and humans have a distinct transcriptome profile, exhibiting an upregulation of gene transcripts associated with the cell stress response, brain inflammation, and age-related diseases such as AD (Lukiw, 2004; Sierra et al., 2007; Galatro et al., 2017; Olah et al., 2018; Bonham et al., 2019). In aging mice, microglia undergo morphological changes, exhibit a less ramified cell morphology, reduced process length, and increased soma volume (Sierra et al., 2007; Tremblay et al., 2012; Hefendehl et al., 2014). These age-related morphological alterations vary across brain regions and have further been correlated with shifts in microglial function (Hart et al., 2012). For example, microglia display an age-diminished response to laser injury, reacting with a drastic decrease in process motility compared to their younger counterparts (Hefendehl et al., 2014). Microglia in aged mice also display increased mitochondrial activity, which may impact neuronal function in aging (Ye and Johnson, 1999; Njie et al., 2012; Ritzel et al., 2015). Aging additionally impaired microglial phagocytic capabilities and lysosome function as illustrated through the accumulation of lipofuscin-like lysosomal inclusions (Njie et al., 2012; Ritzel et al., 2015; Safaiyan et al., 2016). Likewise, microglia can accumulate lipid droplets in age, which is accompanied by defective microglial phagocytosis and an increased production of toxic cell byproducts including reactive oxygen species and pro-inflammatory cytokines (Shimabukuro et al., 2016; Marschallinger et al., 2020; Loving et al., 2021). Microglia that have become dysfunctional, such as dystrophic or senescent microglia, increase with age, have shorter telomeres, and less complex, fragmented processes (Streit et al., 2004; Flanary et al., 2007; Lopes et al., 2008).

Astrocytes in aged mice also display elevated levels of inflammatory and oxidative stress genes (Jiang and Cadenas, 2014; Boisvert et al., 2018; Clarke et al., 2018; Habib et al., 2020). An elevation in genes associated with neuroinflammation accompanied by the loss of an astrocyte’s other normal functions such as synapse regulation characterize reactive astrocytes, which increase with age and can influence the outcomes of brain injury or neurodegenerative diseases (Myer, 2006; Boisvert et al., 2018; Clarke et al., 2018; Reichenbach et al., 2019). In addition, astrocytes isolated from aged mice expressed increased levels of genes in synapse elimination pathways as well as those linked with age-related diseases including *Snca* and *Sncg*, two genes associated with PD (Boisvert et al., 2018; Early et al., 2020; Pan et al., 2020). Similar to microglia, cultured astrocytes isolated from aged mice display an increase in mitochondrial activity, which limits the substrate supply from astrocytes to neurons and can contribute to age-related cognitive decline (Jiang and Cadenas, 2014). Astrocytes in aged mice also mirror aged microglia in that they undergo region-specific morphological changes, demonstrating variable changes in cell complexity, a reduced domain size with short, stubby processes, and decreased astrocyte coupling through gap junctions (Rodríguez et al., 2014; Jyothi et al., 2015; Bondi et al., 2021; Popov et al., 2021). Age-dependent morphological changes were also concomitant with deficiencies in astrocytic physiology, specifically in potassium buffering and glutamate clearance, which impaired synaptic plasticity and hippocampal long–term potentiation (Popov et al., 2021). Taken together, these findings suggest that changes in microglia and astrocyte function due to aging may alter outcomes following brain injury or impact age-associated disease progression, though this continues to be a topic of debate.

## Influences of Microglia and Astrocytes in Neurodegenerative Disease

### Alzheimer’s Disease

In AD microglia and astrocytes undergo functional change, enhancing their neuropathological or neuroprotective influences in disease progression depending on their microenvironment. For example, in human patients with mild cognitive impairment and AD mouse models, microglia can release pro-inflammatory cytokines that induce neuroinflammation, which can exacerbate AD pathology resulting in increased Aβ plaque deposition, neuronal tau accumulation, and synapse loss (Tarkowski, 2003; Yoshiyama et al., 2007; Wright et al., 2013). In depletion studies elimination of microglia through the inhibition of colony-stimulating factor 1 receptor (CSF1R) in 5xFAD mice, an AD mouse model that overexpresses amyloid-β (Aβ) and develops plaque pathology, there was impaired Aβ plaque formation, reduced inflammation, and neuronal loss (Spangenberg et al., 2016, 2019). These results suggest microglia contribute to the development and progression of AD pathology by causing neuronal atrophy and initiating plaque formation among other roles (Spangenberg et al., 2016, 2019). Microglia can also activate the NOD, leucine-rich repeat (LRR), and pyrin-domain containing 3 (Nlrp3) inflammasome, which results in the production of Asc specs that cross-seed Aβ plaques and enhance both amyloid and tau-associated pathology in AD (Heneka et al., 2013; Venegas et al., 2017; Ising et al., 2019). Inhibition of the Nlrp3 inflammasome in AD mouse models was recently shown to reduce Asc speck formation, plaque pathology, and microgliosis, suggesting that Nlrp3 inflammasome activation in microglia could be driving its neurotoxic effects (Lonnemann et al., 2020; Shippy et al., 2020). In contrast, microglia also appear to be neuroprotective early on in AD pathology (Condello et al., 2015; Maphis et al., 2015; Hong et al., 2016; Wang et al., 2016; Yuan et al., 2016). Microglia have been shown to act as a physical barrier between neurotoxic plaques and the surrounding CNS, thereby protecting neurons from damage (Condello et al., 2015; Wang et al., 2016; Yuan et al., 2016). The beneficial contributions of microglia in disease were further highlighted when observing triggering receptors expressed on myeloid cells (TREM2) deficient 5xFAD mice or mice that lack an important microglial receptor (Wang et al., 2015, 2016; Ulland et al., 2017). Microglial deficiencies in TREM2 are associated with increased AD risk in humans and result in diminished microglial responses to amyloidosis in 5xFAD mice (Guerreiro et al., 2013; Jonsson et al., 2013; Wang et al., 2015, 2016; Keren-Shaul et al., 2017; Kleinberger et al., 2017; Krasemann et al., 2017; Zhou et al., 2020). In TREM2 deficient 5xFAD mice, total microglial cell counts were lower and microglia were less likely to associate with and internalize Aβ plaques (Wang et al., 2015, 2016). Microglia in 5xFAD TREM2 knock-out mice also accumulated more autophagic-like vesicles compared to 5xFAD microglia, a trend also verified in human AD patients (Ulland et al., 2017). This microglial deficit was partially rescued through dietary cyclocreatine supplementation, resulting in normalized microgliosis as well as microglial plaque clustering and significantly reduced plaque-associated neurite dystrophy (Ulland et al., 2017). Thus, functional microglia are crucial for eliciting a proper immune response in AD and can either exacerbate or help attenuate associated disease pathology.

Like microglia, astrocytes have also been implicated in roles that exacerbate or are neuroprotective in AD. Astrocytic ApoE_4_ expression, a strong genetic risk factor for late-onset AD, potentiates neuronal tau aggregation in multiple *in vitro* and *in vivo* tau models of AD, prompting AD development (Jablonski et al., 2021). Similarly, astrocytes in primary co-cultures with rat embryo neurons were demonstrated to exacerbate Aβ-induced neurotoxicity, caspase-3 activation, and the production of caspase-3-cleaved tau, which is more likely to aggregate (Garwood et al., 2011). However, the administration of an anti-inflammatory agent, minocycline, reduced tau phosphorylation and other astrocytic inflammatory responses as well as associated neuronal loss, further illustrating the pathogenic contributions of astrocytes to AD (Garwood et al., 2011). Astrocytes also exacerbate AD pathology through their production of hydrogen peroxide and their dysregulated metabolic processes resulting in compromised neuronal support (Oksanen et al., 2017; Chun et al., 2020). In parallel, reactive astrocytes are elevated in localized regions associated with neurodegeneration in human AD patient post-mortem tissue including the hippocampus and prefrontal cortex (Liddelow et al., 2017). This finding mirrors trends seen in other research using human tissue, noting an accumulation of Aβ42, a pathogenic form of amyloid-β peptides, in astrocytes within the entorhinal cortex of clinically diagnosed sporadic AD patients (Nagele et al., 2003). The amount of Aβ42 accumulation in reactive astrocytes was directly correlated to the extent of AD pathology, suggesting that astrocytes contribute to the local inflammatory response (Nagele et al., 2003). In contrast, previous literature has demonstrated that astrocytes have neuroprotective effects in AD. This notion was illustrated through the ablation of reactive proliferating astrocytes in a transgenic AD mouse, which resulted in elevated cortical Aβ pathology and increased levels of monomeric Aβ in brain homogenates (Katsouri et al., 2020). In addition, astrocyte ablation reduced hippocampal neuronal and synaptic density, which was accompanied by increased neuroinflammation and spatial memory deficits (Katsouri et al., 2020). Though astrocytes are known to produce neurotoxic products following systemic injection of LPS or induced ischemia, cytokines released from astrocytes such as TIMP-1 have also been coupled with neuroprotective effects (Zamanian et al., 2012; Liddelow et al., 2017; Saha et al., 2020). For instance, in a well-characterized Aβ-infused rat model of AD intra-cerebroventricular TIMP-1 injection resulted in a reduction in Aβ load and Aβ-induced apoptosis in the hippocampus and cortex (Saha et al., 2020). Furthermore, TIMP-1 treated rats displayed restored synaptic integrity and showed an improvement in memory including associative learning (Saha et al., 2020). Additionally, a subset of reactive astrocytes expressing higher levels of GLT-1, an abundant glutamate transporter, was identified in AD patients that had neuropathological changes consistent with AD but without dementia, unlike AD patients who had dementia (Kobayashi et al., 2018). This finding suggests GLT-1 expressing reactive astrocytes could help preserve cognitive function (Kobayashi et al., 2018). Together, these results indicate that astrocytes are important mediators of neuroinflammation and, depending on the context, can contribute to neurotoxic or neuroprotective responses in AD models.

### Parkinson’s Disease

Similar trends associating glial cells with beneficial and detrimental effects were noted within the context of PD. Microglial reactivity in PD was associated with similar pathological features as those identified in AD such as increased neuroinflammation, which precedes α-syn pathology in PD mice (Marinova-Mutafchieva et al., 2009; Izco et al., 2021). Another study targeting metabotropic glutamate receptor 5 (mGluR5), a G-coupled protein receptor that specifically inhibits the microglial inflammatory response, showed that mGluR5 activation partially inhibits microglial reactivity *in vitro* and had an anti-inflammatory effect *in vivo*, partly protecting neurons from neurotoxicity induced by microglial reactivity (Zhang et al., 2021). Like in aging, microglia in the substantia nigra, a brain region severely affected by PD, of postmortem PD patient brains have been shown to accumulate more neutral lipids or triglycerides compared to healthy controls (Brekk et al., 2020). Brains from patients with PD also contained a higher abundance of microglia in the substantia nigra compared to healthy subjects, suggesting microglia could be reactive (Brekk et al., 2020). Though microglial pathogenicity was not specifically measured in this study, lipid- rich microglia have been associated with functional deficits in microglia that suggest they exacerbate neurodegeneration (Marschallinger et al., 2020). Microglia are also associated with neuroprotective roles in PD, clearing neuronal α-syn and mitigating neuronal degeneration (Choi et al., 2020). Specifically, transgenic PD mice demonstrated that microglia exhibit synucleinphagy in PD, which is the microglial engulfment of α-syn into autophagosomes for degradation through selective autophagy (Choi et al., 2020). When microglial autophagy was disrupted, α-syn mediated neurotoxicity and neurodegeneration were enhanced, demonstrating a protective role for microglia in PD (Choi et al., 2020). Furthermore, in a 1-methyl-4-phenyl-1,2,3,6-tetrahydropyridine (MPTP) induced mouse model of PD, microglial depletion through CSFR1 inhibition aggravated MPTP-induced neurotoxicity, resulting in locomotor impairment and the loss of dopaminergic neurons (Yang et al., 2018). Additionally, the knockdown of microglia-specific Cav1.2, a voltage-dependent calcium channel, in MPTP-induced PD mice resulted in severe degeneration of dopaminergic neurons and accompanying motor deficits, further suggesting microglia help mitigate disease progression (Wang et al., 2019). In all, these and past studies demonstrate that microglia play dual roles in PD, either contributing to or counteracting PD progression (Toku et al., 1998; Zhang et al., 2005).

In PD, astrocytes also play a dual role. For instance, in an α-syn preformed fibril PD mouse model inhibition of reactive astrocytes was neuroprotective (Yun et al., 2018). Specifically, the inhibition of reactive astrocytes protected against dopaminergic neuron loss and associated behavioral deficits *in vivo* (Yun et al., 2018). Likewise, when applied to primary rat astrocyte cultures multiple forms of α-syn, including monomeric, oligomeric, and fibrillar forms, can cause astrocytes to become reactive (Chavarría et al., 2018). Astrocyte reactivity was accompanied by increased levels of intracellular oxidants and pro-inflammatory cytokine release (Chavarría et al., 2018). In co-culture with hippocampal neurons, astrocytes exposed to different α-syn species increased cytotoxicity, provoking neuronal death (Chavarría et al., 2018). Additionally, selective expression of A53T mutant α-syn, a genetic mutation in α-syn linked with increased PD risk, in astrocytes of transgenic PD mice compromised their normal functions as seen through a downregulation of GLAST1 and GLT1, two proteins associated with glutamate transport (Gu et al., 2010). Astrocytic A53T α-syn expression was also associated with neurological dysfunction, a shortened lifespan, and neurodegeneration (Gu et al., 2010). Furthermore, some of these pathological trends were exhibited in induced pluripotent stem cells (iPSC)-derived astrocytes from PD patients with a G2019S mutation in the Leucine Rich Repeat Kinase 2 (*LRRK2*) gene, which is the most common cause of familial PD (Ramos-Gonzalez et al., 2021). iPSC astrocyte cultures from these patients demonstrated decreased homeostatic support to neurons and elevated oxidative stress, an observation that has previously been linked with enhanced neurodegeneration both *in vitro* and *in vivo* (Hashioka et al., 2009; Gu et al., 2010; Chavarría et al., 2018; Ramos-Gonzalez et al., 2021). RNA sequencing of *LRRK2* G2019S iPSC-derived astrocytes also revealed a downregulation of transforming growth factor beta 1 (TGFB1) and matrix metallopeptidase 2 (MMP2), which are both involved in regulating the extracellular matrix, a role that can profoundly alter an astrocyte’s response to inflammatory stimuli (Johnson et al., 2015; Booth et al., 2019). Therefore, astrocytes carrying the *LRRK2* G2019S mutation have a reduced capacity to perform roles contributing to neuroprotection and thus, support PD pathogenesis. Astrocytes in PD have also been associated with providing a neuroprotective potential. Astrocyte-specific overexpression of human DJ-1 also known as Parkinson Disease Protein 7 (PARK7), a redox sensitive protein with multiple reported functions crucial for mitochondrial physiology and protein transcription, was shown to mitigate neurotoxicity in a PD rat model (De Miranda et al., 2018). In rats overexpressing astrocytic DJ-1 there was a reduction in mitochondrial dysfunction, α-syn accumulation and phosphorylation, astrocyte-induced neuroinflammation, and neurodegeneration in dopaminergic neurons in the substantia nigra (De Miranda et al., 2018). Additionally, following the MPTP challenge, mice with astrocytes lacking Kir6.1, an ATP-sensitive potassium channel, exhibited increased dopaminergic neuron loss in the substantia nigra compacta and more severe motor dysfunction compared to controls (Hu et al., 2019). Similarly, astrocytic Kir6.1 deletion resulted in increased production of mitochondrial reactive oxygen species, astrocyte-mediated neuroinflammation, and decreased mitophagy in astrocytes (Hu et al., 2019). Another study using rats injected with 6-hydroxydopamine, which induces dopaminergic denervation of the striatum, revealed that striatal astrocytes possess physiological phagocytic properties (Morales et al., 2017). While this functional response is more typical of microglia, this role in astrocytes may be critical for clearing striatal debris at the onset of PD (Morales et al., 2017). In parallel, iPSC-derived astrocytes from PD patients carrying an *ATP13A2* mutation, which affects lysosome function, were protective, resulting in rapid phagocytosis of α-syn and higher lysosomal degradation rates (Tsunemi et al., 2020). When co-cultured with iPSC-derived dopaminergic neurons, the iPSC-derived astrocytes further prevented neuronal α-syn accumulation and inter-neuronal α-syn transfer (Tsunemi et al., 2020). Collectively, these studies reveal that astrocytes, like microglia, can be either toxic or beneficial in PD pathology.

## Microbiome Influences on Microglia and Astrocyte Function in Health

**Figure 2:**
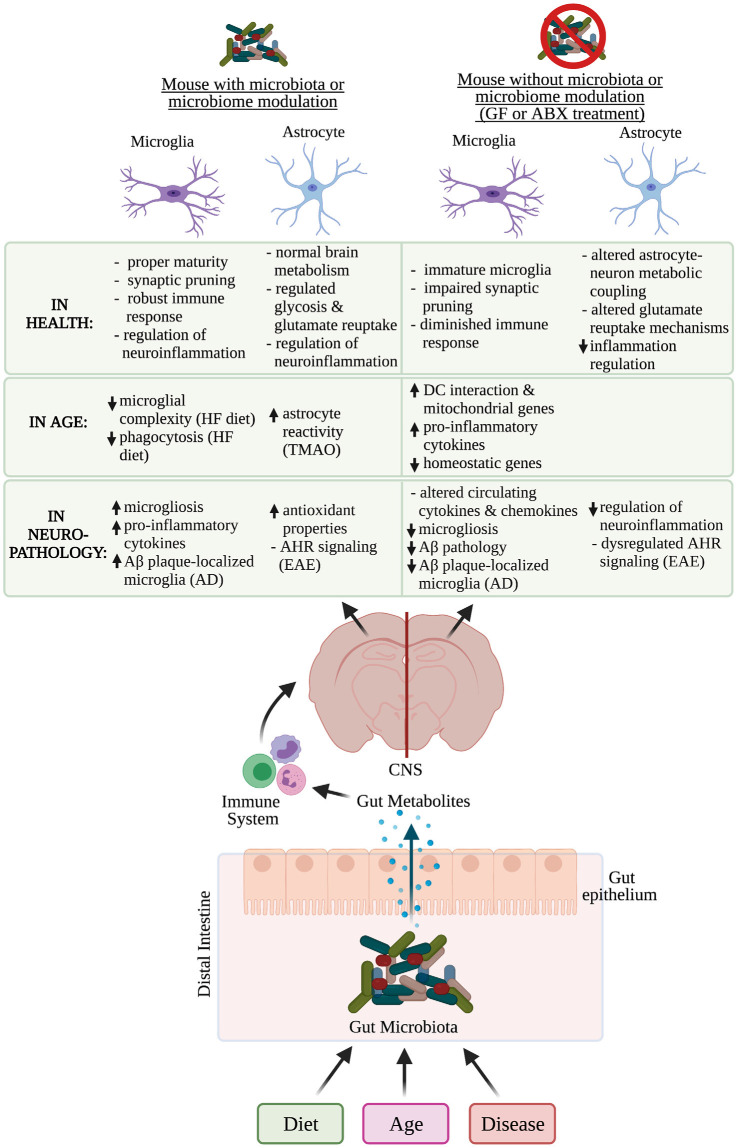
Microbiome influence on microglia and astrocyte function in health, age, and neuropathology. Multiple factors affect an individual’s microbiome composition including diet, age, and disease. In the distal intestine, gut metabolites can cross the gut epithelium and modulate the immune system, which can influence the function of microglia and astrocytes in health, age, and neurodegenerative diseases. Functions of microglia and astrocytes that are altered in mice who have had their microbiome altered, contain a microbiome or lack thereof have been identified. Aβ, amyloid-β; GF, germ-free; ABX, antibiotic; AD, Alzheimer’s disease; EAE, experimental autoimmune encephalomyelitis; CNS, central nervous system; DC, dendritic cell; HF, high-fat; TMAO, trimethylamine N-oxide; AHR, aryl hydrocarbon receptor. *Created with Biorender.com.

Increasingly the bidirectional communication between the brain and the gut, termed the gut-brain axis, has been recognized as playing an important role in brain health and disease. The gut microbiome is predominantly composed of bacteria but it can also contain viruses, fungi, archaea, and helminths (Vemuri et al., 2020). The gut microbiome composition varies across individuals as well as species and is influenced by a variety of factors including age, diet, and disease among others (Figure 2; The Human Microbiome Project Consortium, 2012; David et al., 2014; Nagpal et al., 2018; Carmody et al., 2019; Xu et al., 2019; Manor et al., 2020; Kundu et al., 2021; Wilmanski et al., 2021). Microbial metabolites produced by gut bacteria in humans have been shown to impact host health by directly interacting with immune cells and intestinal epithelial cells, which allows for selective penetration of nutrients while preventing the passage of harmful stimuli such as toxins in subjects with healthy gut barrier function (Figure 2; Cabinian et al., 2018; James et al., 2020; Ghosh et al., 2021). As gut permeability increases with age, certain individual differences in the microbiome may also be causative in exacerbating or ameliorating disease as differentially abundant microbiota and gut metabolites such as trimethylamine N-oxide (TMAO) have been associated with increased risk of AD and other neurogenerative diseases (Man et al., 2015; Qi et al., 2017; Vogt et al., 2017, 2018; Brunt et al., 2021; Romano et al., 2021). In humans, the gut microbiome has been implicated in modulating immune function both systemically and in the CNS, suggesting that the microbiome can influence microglial and astrocyte function (Erny et al., 2015; Buford et al., 2018; Schluter et al., 2020; Sanmarco et al., 2021).

Though the timing of fetal microbiome development is highly debated, convincing evidence suggests the fetus begins developing its microbiome during birth in the mouse and human and is generally compositionally similar to the maternal microbiome (Jiménez et al., 2008; Ferretti et al., 2018; Younge et al., 2019; Kennedy et al., 2021). During early life, the microbiome is critical for postnatal innate immune development as well as microglial maturation, which could affect how microglia respond in physiological and pathological conditions in adulthood (Erny et al., 2015; Gomez de Aguero et al., 2016; Matcovitch-Natan et al., 2016; Thion et al., 2018). The microbiome also plays a role in postnatal microglial synaptic pruning, affecting neurodevelopment outcomes (Lebovitz et al., 2019; Luck et al., 2020). Broadly, the host microbiota is a critical component for shaping microglial function (Erny et al., 2015). Compared to conventionally colonized specific pathogen free (SPF) mice, germ-free (GF) mice lacking a microbiota contain microglia with a more complex morphology and an immature phenotype (Erny et al., 2015). As a result, microglia in GF mice are more prone to functional deficits including a diminished immune response, a finding mirrored in aged mice (Hefendehl et al., 2014; Erny et al., 2015; Ritzel et al., 2015; Galatro et al., 2017). Particularly, *Bifidobacteria* species and *Lactobacillus murinus HU-1* can normalize dystrophic microglia, indicating that microglial function could be modulated by a specific type of bacteria in the postnatal period (Lebovitz et al., 2019; Luck et al., 2020). To date, the impact of the microbiome on astrocyte function in postnatal development remains to be explored. However, there is one study that demonstrated maternal probiotic supplementation with *Lactobacillus acidophilus* and *Bifidobacterium infantis* attenuates astrocyte reactivity in postnatal offspring (Lu et al., 2020). Taken together, these studies establish that microbiome influences on brain function occur early on and could contribute to neurological outcomes in age or disease through adulthood.

In mature animals, the microbiome also influences the homeostatic roles of microglia and astrocyte function in health. The importance of the microbiome in the normal microglial immune response is illustrated by the fact that in GF mice, microglia exhibit a diminished response to bacterial and viral infections, compared to microglia from SPF mice (Erny et al., 2015; Brown et al., 2019). Furthermore, the depletion of the microbiome using broad-spectrum antibiotics in 6–10 week old SPF mice significantly changed microglial morphology (Erny et al., 2015). After antibiotic administration, microglia in SPF mice resembled malformed microglia similar to those identified in GF mice, suggesting that the microbiome is critical for microglial homeostasis in physiological conditions (Erny et al., 2015). Although there is limited research illustrating how the microbiome directly modulates other functional roles of microglia, one study links isoflavones and lignans, two classes of highly gut and BBB permeable polyphenol microbial metabolites, with decreased nitric oxide and pro-inflammatory cytokine levels in murine microglia (Johnson et al., 2019). This finding indicates that gut-microbial-derived-metabolites can dampen microglia-specific neuroinflammation and could be beneficial to administer as a therapeutic for neurodegenerative diseases.

In addition to microglial changes, the microbiome was also shown to be important for maintaining astrocyte function. Following gut microbiota perturbations, an upregulation in genes associated with astrocyte-neuron metabolic coupling was detected within the mouse hippocampus (Margineanu et al., 2020). Specifically, there was an upregulation in Pfkfb3 and Atp1a2, two astrocytic proteins responsible for regulating glycolysis and glutamate reuptake, respectively, indicating that the microbiome can affect astrocytic contributions to brain energy metabolism (Margineanu et al., 2020). Additionally, the application of neuroprotective metabolites including sodium butyrate and indole-3-propionic acid to human primary astrocyte cultures showed that both bacterial metabolites could prevent LPS-induced inflammation and mitigate pro-inflammatory cytokine release following an LPS challenge (Garcez et al., 2020). There is also some evidence suggesting that the microbiome impacts processes that microglia and astrocytes are associated with, including adult hippocampal neurogenesis (Ogbonnaya et al., 2015). Likewise, the microbiome influences pro-inflammatory mechanisms within the hippocampus, fear extinction learning, and BBB permeability, all of which microglia or astrocytes are implicated in, through the immune system *via* monocytes or other microbiota-derived signals (Braniste et al., 2014; Campos et al., 2016; Möhle et al., 2016; Chu et al., 2019). Taken together, these findings demonstrate that the microbiome is important for maintaining the homeostatic functions of microglia and astrocytes.

## Microbiome Influences on Microglia and Astrocyte Function in Aging

The microbiome also displays compositional differences with age but work exploring the effects of an aging microbiome, outside of a disease context, on glia remains limited. However, the effects of a short-term high-fat diet on microglia in the amygdala given to young and old rats was recently investigated, paralleling microglial changes identified in aging studies alone (Spencer et al., 2019). Upon receiving the high-fat diet, microglia in the amygdala in aged rats displayed reduced microglial complexity and suppressed phagocytic capacity compared to their younger counterparts (Spencer et al., 2019). Additionally, transcriptional analysis on microglia isolated from aged mouse brains with or without antibiotic-induced gut dysbiosis revealed that aged groups in both treatment conditions were enriched with gene pathways involved in dendritic cell interaction and macrophage cytokine production (Golomb et al., 2020). Microglia from aged mice in both cohorts also exhibited an upregulation of mitochondrial genes and a decreased expression of microglial homeostatic genes such as *Trem2* (Golomb et al., 2020). Though there were similar trends in both the antibiotic-treated and non-antibiotic-treated aged cohorts, microglia with these properties were slightly more prevalent in aged mice receiving antibiotics (Golomb et al., 2020). Together, this data suggests that gut dysbiosis increases the likelihood of developing neuroinflammation during aging.

Similarly, shifts in astrocyte function have been linked with microbiome modulations in aged mice and humans. In a recent study exploring the potential role of TMAO in modulating neuroinflammation and cognitive function, both middle-aged to older adults (65 ± 7 years old) and aged mouse plasma TMAO levels were higher compared to their younger counterparts (Brunt et al., 2021). Likewise, circulating plasma TMAO levels were inversely correlated with working and episodic memory as well as fluid cognition in humans as measured through the NIH Toolbox Cognition Battery Test (Brunt et al., 2021). TMAO levels in 27-month-old mice were also increased in the brain compared to 6-month-old mice, suggesting that TMAO crosses the BBB, and is associated with increased neuroinflammation as well as astrocyte reactivity (Brunt et al., 2021). Furthermore, young mice supplemented with TMAO exhibited similar impairments in memory and spatial learning as observed in aging compared to young control mice (Brunt et al., 2021). In total, these findings suggest elevated TMAO levels that accompany aging are correlated with astrocyte reactivity and increased neuroinflammation associated with cognitive impairments (Brunt et al., 2021). Another approach isolated the microbiome in young and aged mice to broadly explore the effects of age on the microbiome and brain (Lee et al., 2020). In this study, fecal transplant gavages from aged or young SPF mice into young GF mice revealed that the aged microbiome alone was sufficient to produce a cognitive decline in the recipient mice who exhibited depressive-like behavior as well as impaired short–term and spatial memory (Lee et al., 2020). Though this approach did not directly implicate microglia or astrocytes specifically, it was an elegant study design illustrating that the aging gut microbiome impacts the brain and can influence cognitive performance. More research needs to be done to better understand how an aging microbiome directly or indirectly impacts both microglia and astrocyte functions and what that may mean for human health.

## Microbiome Influences on Microglia and Astrocyte Function in Neurodegenerative Disease

The microbiome can also modulate microglia and astrocyte function in neurodegenerative disease as explored in the context of AD and PD mouse models. For example, comparisons of gut microbiota in APP/PS1, an early-onset AD mouse model with Aβ pathology, to wild-type mice spanning 1–9 months old demonstrated distinct changes in bacterial taxa that preceded the development of key features indicative of AD pathology (Chen et al., 2020). The divergence in microbiota composition between wild-type and APP/PS1 mice was noted at 1–3 months of age, before amyloid deposition and plaque-localized microglial activation are apparent (Chen et al., 2020). In addition to reporting decreased microbial richness and diversity in older mice, this study also demonstrated that 6 and 9 months old mice had increased abundances of inflammation-related bacterial taxa (Chen et al., 2020). Microbial products such as short chain fatty acids were also recently identified as modulators of microglial function *in vivo*, increasing microglial reactivity and hindering microglial phagocytosis (Colombo et al., 2021). Likewise, microbiome modulations in 5xFAD mice were shown to differentially control microglial Aβ clearance mechanisms and influence plaque deposition (Mezö et al., 2020). In both GF and antibiotic-treated 5xFAD mice, there was less microglial accumulation around compact Aβ plaques and attenuated plaque pathology in the hippocampi compared to SPF 5xFAD controls (Mezö et al., 2020). These findings are congruent with previous studies that administered an antibiotic cocktail to transgenic APP/PS1 AD mice and showed a reduction in Aβ plaque pathology and plaque-associated microgliosis as well as increased cytokine and chemokine circulation (Minter et al., 2016, 2017; Harach et al., 2017; Dodiya et al., 2019). Though recent literature notes that the reduced plaque deposition trends previously established in two transgenic APP/PS1 mice were only replicable when administered an antibiotic cocktail and that individual antibiotic did not affect cerebral Aβ amyloidosis (Dodiya et al., 2020). AD pathology was also curtailed in 5xFAD mice through the dietary supplementation of β-hydroxybutyrate (BHB), a ketone body produced during ketogenesis, which reduced Aβ plaque formation, microgliosis, and Asc speck formation (Shippy et al., 2020). Similarly, the colonization of a curli-producing gut bacteria, *Escherichia coli* (*E. Coli*), in mice overexpressing α-synuclein, a hallmark of synucleinopathies such as PD, was demonstrated to exacerbate disease progression (Chen et al., 2016; Sampson et al., 2020). Curli are microbial amyloid proteins expressed specifically by gut bacteria, which is not to be confused with amyloidogenic proteins found within the brain such as amyloid-β found in AD plaques (Barnhart and Chapman, 2006). Gut-associated microbial amyloid produced by *Pseudomonas aeruginosa* was recently shown to exacerbate Aβ amyloidosis (Javed et al., 2020). For instance, mice receiving a mono-colonization of amyloid-producing *E. Coli* showed significantly impaired motor and gastrointestinal performance compared to mice with a complex microbiota lacking the amyloid-producing strain (Sampson et al., 2020). Morphometric analysis of midbrain microglia in mice harboring the amyloid-producing strain further displayed concomitant alterations indicative of increased inflammation in the midbrain (Sampson et al., 2020). Using *in vitro* assays, another study illustrated that chlorogenic acid (CGA), a coffee component with antioxidant properties, significantly suppressed the release of inflammatory products from primary microglia and prevented microglia-induced neurotoxicity in dopaminergic neurons (Shen et al., 2012). Collectively, these studies suggest that microbiome products or perturbations can alter microglia and therefore, neurological outcomes in a disease.

While the impact of the gut microbiome on astrocyte function in neurodegenerative disease is not well understood, a few reports using Multiple sclerosis (Ms) and PD mouse models have begun to establish this connection. In experimental autoimmune encephalomyelitis (EAE), a common mouse model of Ms, gut metabolites derived from dietary tryptophan (Trp) were shown to activate astrocytic aryl hydrocarbon receptor (AHR) signaling, which limits NF-κB signaling and suppresses CNS inflammation (Rothhammer et al., 2016). The favorable effects of dietary Trp were observed in wild-type and GFAP-AHR knock-out EAE mice fed a tryptophan-depleted diet (TDD), which both had increased EAE clinical scores, indicative of disease progression (Rothhammer et al., 2016). However, EAE scores could be improved through Trp supplementation in wild-type but not GFAP-AHR knock-out EAE mice, suggesting that Trp-derived ligands of AHR signaling function through astrocytes and serve a beneficial role in hindering disease progression (Rothhammer et al., 2016). AHR signaling mechanisms established in the EAE model were also verified in human brain samples from individuals with Ms, suggesting this signaling mechanism has some clinical relevance and could contribute to Ms pathogenesis (Rothhammer et al., 2016). Microbial-derived tryptophan metabolites were also shown to influence astrocyte function indirectly through microglia (Rothhammer et al., 2018). Specifically, administration of a TDD modulated AHR signaling in microglia, impacting microglial *VEGF-B* and *TGFα* expression, which in turn regulated astrocytic pathogenic responses in EAE *via* NF-κB signaling (Rothhammer et al., 2018). Microglial control of astrocytes *via* VEGF-B and TGFα were further verified in human samples and found to contribute to Ms pathogenesis (Rothhammer et al., 2018). Indirect effects of the microbiome on astrocytes have also been observed in a subset of astrocytes expressing TRAIL, a gene that can induce the apoptosis of other cells (Sanmarco et al., 2021). Gut microbiota depletion *via* antibiotics in SPF *Ifng^EYFP^* reporter mice, a mouse model that fluorescently labels the pro-inflammatory cytokine interferon gamma (IFN-Γ), reduced TRAIL^+^ astrocytes relative to mice receiving a vehicle or an antibiotic treatment followed by a fecal microbiota transfer (Sanmarco et al., 2021). With lower TRAIL^+^ astrocytes, the reporter mice were unable to limit CNS inflammation and therefore, succumbed faster to EAE (Sanmarco et al., 2021). Additionally, PD mice receiving a fecal microbiota transplant (FMT) from healthy control mice exhibited less astrocyte and microglial reactivity (Sun et al., 2018). PD mice that regularly received coffee components, caffeic, acid and CGA, also displayed enhanced antioxidative properties in striatal astrocytes and exhibited less neurodegeneration of dopaminergic and intestinal enteric neurons (Miyazaki et al., 2019). Though the current literature is limited and the results here do not comprehensively explore the impact of the microbiome on glia in neurodegenerative diseases, these data indicate that the microbiome can favorably or detrimentally modulate neurodegeneration. This data also presents a promising new frontier to explore therapeutic interventions that hinder disease progression through the modification of the microbiome.

## Summary

Overall, microglia and astrocytes, two immunocompetent glial cells that normally perform vital support roles in the brain, are crucial for responding to injury or pathogenic insults. However, factors including one’s microbiome composition, age, and neurodegenerative disease can alter their physiological functions. By exploring how inflammatory processes or functions of microglia and astrocytes can be manipulated or altered through the microbiome, during aging, and in neurogenerative diseases, we can gain more insight into potential disease mechanisms and discover methods to better regulate age-and disease-associated pathology.

## Concluding Remarks

Though glial cells were initially identified in the very early years of neuroscience research, we have only recently begun to grasp the full scope of glial contribution to brain health, aging, and disease. Although the function of both microglia and astrocytes are dramatically impacted in aging and neurodegenerative disease, it remains unclear whether these functional changes are detrimental or beneficial. Complex interactions between the gut microbiome and brain in health, aging, and disease have also been shown to modulate glial function although literature addressing how the microbiome affects astrocyte function remains especially limited. To better understand the contribution of glia to age and disease, new *in vivo* and *in vitro* techniques need to be employed. Novel, promising approaches including 2Phatal and the use of tri-cultures supporting neurons, microglia, and astrocytes may enable such opportunities, allowing multi-cellular interactions to be observed *in vivo* and *in vitro* (Goshi et al., 2020; Guttikonda et al., 2021). Furthermore, the recent development of an *in vitro* all-human physiomimetic model has exciting implications for how we understand neurodegenerative diseases, broadening the questions that can be asked about how microglia and astrocytes are impacted by or contribute to aging and disease (Trapecar et al., 2021). This model links three complex microphysiological systems including the gut/immune, liver/immune, and cerebral/immune systems in a common culture media containing circulating immune cells in continuous coculture (Trapecar et al., 2021). Using an *in vitro* human physiomimetic model enables a controlled systems approach that will allow organ-organ and organ-immune system interactions mimicking *in vivo* like behavior to be explored. These techniques utilizing multiple cells and systems can more realistically recapitulate *in vivo* inflammatory responses that will allow us to further expand the questions that can be asked and knowledge that can be collected about microglia and astrocytes in the next decade.

## Author Contributions

KH conceived the review. KH, KM, and TU discussed and contributed to the writing of this review. All authors contributed to the article and approved the submitted version.

## Conflict of Interest

The authors declare that the research was conducted in the absence of any commercial or financial relationships that could be construed as a potential conflict of interest.

## Publisher’s Note

All claims expressed in this article are solely those of the authors and do not necessarily represent those of their affiliated organizations, or those of the publisher, the editors and the reviewers. Any product that may be evaluated in this article, or claim that may be made by its manufacturer, is not guaranteed or endorsed by the publisher.
